# The Attitude of Great Writers Towards Disease

**Published:** 1892-11-26

**Authors:** 


### The Attitude of Great Writers Towards Disease.

### XVI.—BOCCACCIO.

Visitors to Florence will remember the steep pathway set between high stone walls, which leads from the city towards the crest of Fiesole. Those white walls, scored with the old winding Etruscan markings block out the prospect very provokingly. Only now and then at a sudden turn there is a glimpse to be had of the brown roofs and towers behind, of the gold cross of the Duomo gleaming against the soft, deep blue above, and of Giotto's Tower, the lily of architecture, shining with new graces from every new point of view. Over the walls rises the grey green foliage of some olive taller than the rest, or, perhaps, a tangle of roses drops down far overhead, for June in Florence brings many an echo of the line, "'Twas roses, roses all the way." Sometimes a half-opened doorway by the roadside discloses the rich fertility of the soil where "corn and wine and oil" grow side by side; the vines stretching their young tendrils across from tree to tree, while the maize stands thick between the rows. It is but a short distance along this road before the villa is reached, memorable as lying in the very gardens where Boccaccio's young men and maidens took refuge from the pestilence. Here, from this very spot where the pomegranate and myrtle, the orange flowers and roses, were blooming then as now, the little company looked down at mid-day on the doomed city, lying in a deadly haze under a pitiless sky, where in four months no fewer than 100,000 had perished of the plague. Then, as now, the four bridges of the winding Arno met their view, the unfinished Duomo with the still older Baptistery, Giotto's Campanile, and many a fair church still standing and many a mighty palace framed to withstand the siege of enemy or neighbour. It was turning from this very sight, set round in those low clustering hills, that Dioneo pleaded with a sick shuddering that either they should distract their minds with song and jest or else that he might be suffered to return thither, where he had left all his thoughts behind, in the city of tribulation.

Let us leave them here to tell their tales of love and satire and scandal, and free their minds from the horrible atmosphere of disease and death, seated among the soft grass, in a spot where the high myrtle hedges exclude every ray of sun, where no sound is heard but the voice of the cicala above in the olives and the soft plash of the fountain, and let us descend to the doomed city and learn what Boccaccio has to tell us of the source and progress of the disease which had left them friendless and orphans in this vivid juxtaposition of gaiety and despair.

And first we learn that this plague, which visited Florence in the year 1348, had been long expected. Some years before it had commenced its ravages in the East, and had crept steadily nearer, until, at its first appearance in Europe, hasty sanitary precautions were adopted by the various cities in ts path, with the hope of averting the danger. Florence was purified of many abominations by officials specially appointed for that purpose; stringent rules were enforced against admitting any sick persons within the gates, and much advice was circulated about the best method of maintaining health. It would be interesting to know in what the advice consisted. One curious precaution is betrayed in the desire of the little company of fugitives to keep themselves awake all day by their tales, since it was deemed unhealthy to sleep in the daytime. Some of the inhabitants placed their trust in a temperate life, retired in the strictest seclusion, and nourished on the most delicate food procurable, and the choicest wines. It may be suspected that the water was almost universally contaminated, and that therefore some show of reason lay with those who trusted to wine alone as a specific for the disease.

No precautions availed, and about Easter time the first cases occurred, followed by a period when every attempt to cope with the infection broke down hopelessly, and the plague raged like a conflagration. It was noted that the symptoms varied from those of the Eastern plague. The fatal sign was no longer a bleeding at the nose, but the appearance of a boil or tumour (gavocciolo), and later on in the epidemic of black or livid spots on the skin. After the appearance of either of these death ensued within three days without fever or other mark of illness. Neither drugs nor doctors availed in the smallest degree to procure relief, though the number of those professing a cure, both men and women, increased to a prodigious extent. Many of these had not the slightest knowledge of medicine.

Much terror was caused by the rapidity with which the disease was communicated by contact, or by the medium of clothes, and even animals. It is quite evident that the most ordinary precautions were unknown, and the following scene, witnessed by Boccaccio himself, gives a curious picture of the sanitary condition of the streets. It seems the belongings of some poor wretch who had recently died were flung into the public street. There Boccaccio noticed two pigs rooting and tearing at the dead man's clothes, and affirms that in an incredibly short space of time, while he watched, the animals staggered as though taken with sudden poison and fell down dead among the rags.

Spices, herbs, or flowers were commonly carried about to mitigate the foul odours with which the city began to be filled. For the dead were frequently left unburied in the streets for lengthened periods, or dying in their own houses, remained there undiscovered, and the fumes of the various drugs in use seemed to have been hardly less horrible.

In the midst of all this it is not wonderful that something like a general panic should have ensued. The officers of the law were mostly ill or dead. No business of any kind was carried on. People lived indiscriminately in each others houses, passing from one to another at will, and spending their days in aimless wanderings to and fro. Many fled to different parts of the country abandoning those dearest to them without remorse. The sick whose own relatives deserted them were in evil case. They were dependent on the offices of hired attendants, who not unnaturally demanded exorbitant pay, and seldom did more than place food and drink within reach of the patients and watch them die. It was noted as a relaxation of the morals of the times that the noblest ladies were frequently reduced to the ministration of male attendants. It is certain that these nurses very usually fell victims themselves to their desire for pay or plunder.

Before long the outlying parts of the country became infected by the fugitives, and the peasants died by thousands on the wayside and in the fields. All agricultural work was abandoned, and the crops remained unreaped. It was remarked that the cattle, finding no care bestowed on them, went out to pasture daily as usual, and returned to their stalls at the close of day without deviating from the ordinary routine.

The priests alone, although in rapidly diminishing numbers, seem to have stuck to their posts. The work of burial, so far as it rested with them, was performed with decency, even at the height of the panic, and some form of religious service seems never to have been lacking. It was at the celebration of High Mass at Santa Maria Novella that the seven maidens and three cavaliers encountered each other and agreed to allow themselves a ten days' respite from the horrors surrounding them. It seems likely that the plague was even then abating. Had it been otherwise it is scarcely probable that the little company should, at the end of their short sojourn, have unanimously agreed to forsake their safe and pleasant refuge for the deadly atmosphere of the city. A terrible home coming it cannot have failed to be.

"Oh, how many grand palaces, how many beautiful houses, how many noble habitations, at one time filled with goodly families, with lords and ladies, remained empty even to the lowest of the servants! Oh, how many memorable families, what ample inheritance, what famous riches, seemed likely to remain without an heir! How many valorous men, how many beautiful women, how many lovely children, whom (to say nought of others) Galen, Hippocrates, or Æsculapius would have deemed entirely healthy, dining in the morning with parents, companions, and friends were that very evening to sup with those who had passed away!"

